# Bases for Treating Skin Aging With Artificial Mitochondrial Transfer/Transplant (AMT/T)

**DOI:** 10.3389/fbioe.2020.00919

**Published:** 2020-08-13

**Authors:** Micaela Balcázar, Stalin Cañizares, Tatiana Borja, Patricia Pontón, Sirivanh Bisiou, Eva Carabasse, Angela Bacilieri, Celia Canavese, Ramiro F. Diaz, Francisco Cabrera, Andrés Caicedo

**Affiliations:** ^1^Escuela de Medicina, Colegio de Ciencias de la Salud COCSA, Universidad San Francisco de Quito USFQ, Quito, Ecuador; ^2^Servicio de Patología, Hospital Voz Andes, Quito, Ecuador; ^3^CEDIA-USFQ Research Initiative, Corporación Ecuatoriana para el Desarrollo de la Investigación y Académica CEDIA and Universidad San Francisco de Quito USFQ, Quito, Ecuador; ^4^Université de Montpellier, Faculté de Medicine, Montpellier, France; ^5^Escuela de Medicina Veterinaria, Colegio de Ciencias de la Salud COCSA, Universidad San Francisco de Quito USFQ, Quito, Ecuador; ^6^Instituto de Investigaciones en Biomedicina, Universidad San Francisco de Quito USFQ, Quito, Ecuador; ^7^Mito-Act Research Consortium, Universidad San Francisco de Quito USFQ, Quito, Ecuador; ^8^Sistemas Médicos SIME, Universidad San Francisco de Quito USFQ, Quito, Ecuador

**Keywords:** skin, aging, senescence, mitochondria, artificial mitochondria transfer transplant (AMT/T), MitoCeption, regenerative medicine

## Abstract

The perception of mitochondria as only the powerhouse of the cell has dramatically changed in the last decade. It is now accepted that in addition to being essential intracellularly, mitochondria can promote cellular repair when transferred from healthy to damaged cells. The artificial mitochondria transfer/transplant (AMT/T) group of techniques emulate this naturally occurring process and have been used to develop therapies to treat a range of diseases including cardiac and neurodegenerative. Mitochondria accumulate damage with time, resulting in cellular senescence. Skin cells and its mitochondria are profoundly affected by ultraviolet radiation and other factors that induce premature and accelerated aging. In this article, we propose the basis to use AMT/T to treat skin aging by transferring healthy mitochondria to senescent cells, possibly revitalizing them. We provide insightful information about how skin structure, components, and cells could age rapidly depending on the amount of damage received. Arguments are shown in favor of the use of AMT/T to treat aging skin and its cells, among them the possibility to stop free radical production, add new genetic material, and provide an energetic boost to help cells prolong their viability over time. This article intends to present one of the many aspects in which mitochondria could be used as a universal treatment for cell and tissue damage and aging.

## Introduction

Mitochondria are organelles found in eukaryotic cells that have an important role in the maintenance of homeostasis and tissue health. Mitochondria can produce up to 95% of all ATP in the cell and sustain metabolic processes such as the tricarboxylic acid cycle (TCA), beta-oxidation, calcium regulation, protein synthesis, among others (McCormack et al., 1990; He et al., 2012; Tzameli, 2012; Birsoy et al., 2015). They also play a major role in inducing cell differentiation, sustaining proliferation, maintaining cell survival, and triggering apoptosis, all of which are essential for the development and maintenance of an organism in time (Vakifahmetoglu-Norberg et al., 2017; Noguchi and Kasahara, 2018). Mitochondrial dysfunction increases as age progresses. This organelle accumulates damage with age and through the constant exposure to external factors, like ultraviolet radiation (UVR), which harms its function and genetic patrimony (Naidoo et al., 2018; Cabrera et al., 2019). The presence of dysfunctional mitochondria has been associated with neurological and degenerative disorders, obesity, type 2 diabetes, and cancer (Angelova and Abramov, 2018; Porporato et al., 2018; Cantó, 2019).

The skin is the largest organ in the human body, serving as our connection to the environment and protecting us from diverse stressors. A variety of environmental factors affect the skin, inducing either a positive or negative response. Ultraviolet radiation induces positive changes in our physiology through skin sensing and reactions (Slominski A. et al., 2012). Other factors, such as pollutants and microbial insults, contribute to skin aging and decrease the functionality of the skin (Bocheva et al., 2019). The neuroendocrine messengers produced after UVR exposure include neuropeptides, biogenic amines, serotonin, and melatonin. These stimulate the hypothalamus, pituitary gland, and adrenal glands (HPA axis), causing effects in our mood, immune system, and behavior (Slominski et al., 2018b). In skin cells, UVR induces the release of neuro-endocrine-immune messengers into the circulation to maintain homeostasis, leading to melanogenesis and keratinocytes proliferation (El-Abaseri et al., 2006; Slominski A. et al., 2012; Slominski et al., 2018b). The skin mitochondria play an important role in supporting these processes, as they are an essential factor in the maintenance of skin health (Slominski A. et al., 2012; Slominski et al., 2015a, 2017). Excess of UVR, pollutants, microbial insults, and wounds can accumulate with time in the skin, causing loss of functionality and cosmetic appearance (Slominski A. et al., 2012; Birch-Machin et al., 2013; Hudson et al., 2016). Skin and its cells’ mitochondria are highly susceptible to detrimental environmental factors. Ultraviolet radiation causes mitochondrial DNA (mtDNA) deletions, perturbing the electron flow, and energy production (Krutmann and Schroeder, 2009). Treating mitochondrial damage would help to mitigate the cumulative damages caused by environmental factors.

Mutations related to monogenic mitochondrial disorders can cause fragmentation of the mitochondrial network in the cell. Affecting this network hampers its capacity to maintain mtDNA stability. When good and damaged mitochondria are unable to fuse within networks, they can’t exchange healthy mtDNA or get rid of damaged DNA copies. This ultimately leads to dysfunctions in the cell and premature senescence (Koopman et al., 2012). For instance, patients with fibromyalgia suffer from oxidative stress and inflammation of the skin which has been linked to mitochondrial dysfunction (Sánchez-Domínguez et al., 2015). Healthy skin depends on the maintenance of functional mitochondria, which could be a target for the development of medical and cosmetic anti-aging treatments.

To our knowledge, there is no effective treatment available to the public to reverse skin aging by targeting mitochondria. The few existing therapeutic options focused on the mitochondria are under development and still, need further *in vitro* assays and clinical validation (Krutmann and Schroeder, 2009; US4603146A, 2019 – Methods for retarding the effects of aging of the skin – Google Patents; Ashrafi and Schwarz, 2013). In addition, no available products, including topical application of natural substances and antioxidants, offer a substantial recovery from many skin aging symptoms such as mtDNA instability, respiration, collagen production, neovascularization, and localized inflammation (Krutmann and Schroeder, 2009).

In this hypothesis article we present the idea and arguments of using the artificial mitochondria transfer/transplant (AMT/T) technique as a possible skin anti-aging therapeutic. We interpret recent data and findings regarding the use of the AMT/T to repair cells and tissue damage. It has been observed previously that the use of AMT/T *in vitro*, *in vivo*, and clinically promotes cell and tissue recovery in different diseases, with effects that could be used to repair skin damage. For example, MitoCeption, one of many AMT/T techniques, induces cell proliferation, migration, and increased respiratory ATP production, processes needed to repair the damage in aged skin (Caicedo et al., 2015). PAMM MitoCeption (Primary Allogeneic Mitochondria Mix Transfer by MitoCeption) repaired UVR damaged cells by recovering the loss of metabolic activity, mitochondrial mass, mtDNA sequence stability in addition to decreasing p53 expression (Cabrera et al., 2019). Published data provided evidence that syngeneic or allogeneic AMT/T transfer of mitochondria was not immunogenic, and additionally that AMT/T to proinflammatory cells, such as TH17, induced cell conversion to Tregs and promoted immune regulation (Luz-Crawford et al., 2019; Ramirez-Barbieri et al., 2019). Beyond *in vitro* applications, AMT/T showed to have regenerative effects *in vivo*, in diseases such as heart and brain ischemia. AMT/T applied clinically to pediatric patients with myocardial dysfunction has also shown positive results on ischemic injured tissues (Liu et al., 2014; Cowan et al., 2016; McCully et al., 2016; Emani et al., 2017). Our hypothesis regarding AMT/T as an antiaging skin therapeutic could be tested *in vitro*, *in vivo*, and clinically, to promote the applications of this technique. The possibility to transfer new mitochondria to senescent or age-induced harmed cells in the skin could represent a plausible option to treat the effects of aging.

## Skin Aging and Mitochondria

Cutaneous aging is a complex and continuous biological process that depends on intrinsic (genetic) and extrinsic factors. These factors contribute to approximately a 50% alteration in skin function when arriving to midlife (WHO, 2009). Physiologically, the skin undergoes constant changes, with the capacity to repair and renovate its constituents after damage. Despite this, environmental factors, biological and chronological aging, all hamper the skin’s abilities to maintain homeostasis and repair.

**FIGURE 1:**
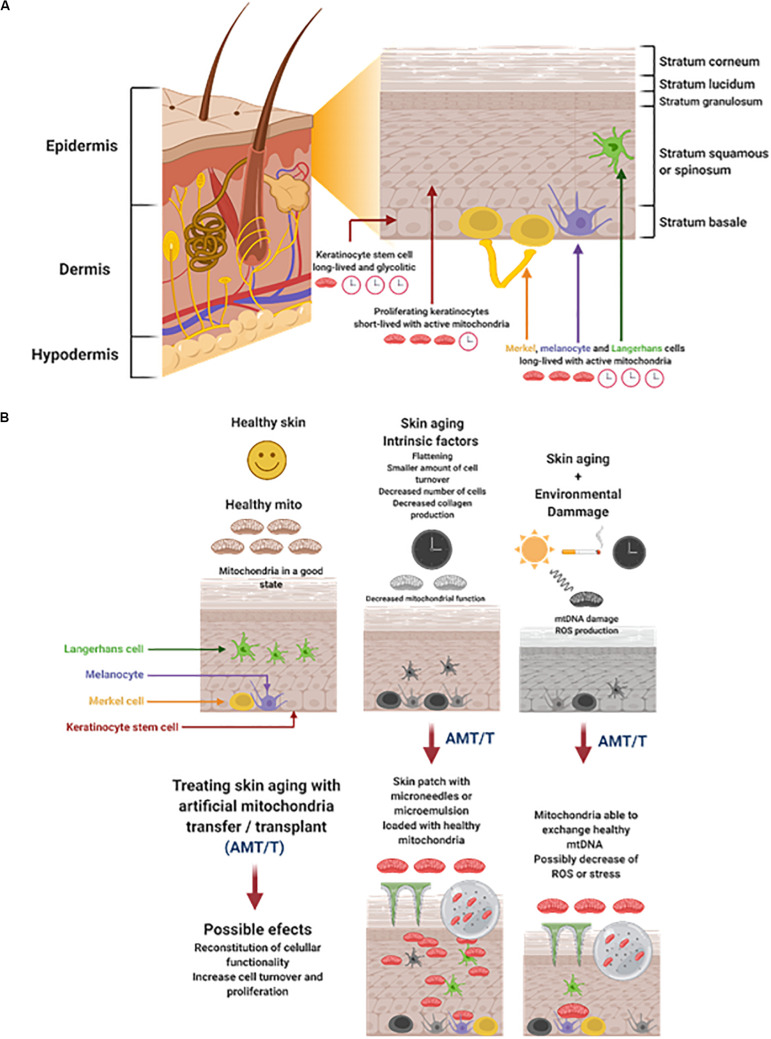
**(A)** Skin structure, cells, and energetic status. The skin is divided in three major cell layers: epidermis, dermis, and hypodermis. The epidermis is the outer layer composed by cells derived from basal stem cells keratinocytes that differentiate, proliferate, and migrate to the surface. The Keratinocytes stem cells are long-lived with a glycolytic metabolism. The proliferating keratinocytes are short-lived with active mitochondria. The Merkel, melanocyte, and Langerhans cells are long-lived and highly dependent of mitochondria function. **(B)** Cutaneous aging is triggered by intrinsic and extrinsic factors that could be treated with AMT/T Skin aging results in the flattening of cell layers and a decrease on cell turnover. This process is accompanied by the decrease of mitochondria function. Time and the environmental damage accumulates, worsening the loss of skin functionality greatly affecting the mitochondria, generating mtDNA mutations, and the increase of ROS production. It would be possible to treat skin aging by AMT/T administered by microneedles or microemulsions. Mitochondria can be delivered to the epidermis possibly resulting in the reconstitution of cellular functionality cell turnover and proliferation. Figure created with BioRender.com.

Genetic or intrinsic features that trigger aging are inevitable. It is possible to observe histologic changes that are subtle when we become old, but with the years the loss of function becomes evident. Histologically, skin aging results in a flattening of the dermoepidermal junction, a smaller amount of epidermal cell turnover, and a decrease in the number of Langerhans cells with advance age (Figures 1A,B) (de Araújo et al., 2019; Fernandez-Flores and Saeb-Lima, 2019). The dermis becomes atrophic because it has a less dense intercellular matrix caused by a decrease in the number of fibroblasts and collagen fibers. As time goes on, collagen fibers become thinner, and after 50 years the thinner fibers start to fragment and progressively experience lysis. This also occurs with elastin fibers, but at a different rate (Yaar et al., 2002).

There is evidence of decreased collagen production in the skin, correlating it with the severity of UVR damage and other factors (Figures 1A,B). In the papillary dermis, a loose meshwork of type I and type III collagen fibers has been observed, where type III collagen becomes more abundant in the adventitial dermis (Meigel et al., 1977; Mills, 2012). Immunofluorescence studies have shown an increase of collagen I and a decrease of collagen III during the aging process (de Araújo et al., 2019). As an individual ages, the dermis gradually replaces the loose meshwork of collagen (type III) with well-distributed dense collagen fibers (type I) (Yaar et al., 2002).

A 10 to 20% reduction of melanin per decade has been observed. Is not clear if this reduction is a consequence of a decreased number or a loss of function of melanocytes (Figures 1A,B) (Rittié and Fisher, 2015). Extrinsic factors including, but not limited to, sun exposure, cigarette exposure, bad nutrition, and low water intake have been seen to induce a process called elastosis. During this processes many aging processes are observed including epidermal atrophy, collagen and elastin fiber fragmentation, and elastin deposition by histopathology (Khavkin and Ellis, 2011). These extrinsic factors, in addition to the loss of collagen, decreases the overall strength of the skin, favors wrinkle formation, and creates a microenvironment that facilitates tumorigenesis and progression (de Araújo et al., 2019).

The skin joins together various systems with different energy demands to perform its protective function (Eckhart et al., 2019). The immune, pigmentary, epidermal, dermal, vascular, and adnexal systems are together and interact in the skin (Slominski et al., 2017). Here, the mitochondria plays a major role in providing the energy support necessary to maintain all systems working. Mitochondria function in the skin could be divided into three major categories: energy, homeostasis, and growth (Stout and Birch-Machin, 2019). Energy consumption and metabolic activity could be classified as proposed by Eckhart et al. (2019), suggesting long-lived stem cells with a low metabolic activity, short-lived differentiating cells with a high metabolic activity (such as keratinocytes), and long-lived differentiated cells with high metabolic activity such as Merkel cells (Eckhart et al., 2019). Interestingly, epidermal keratinocyte stem cells are maintained in a regular number throughout life (Giangreco et al., 2008) and depend on anaerobic glycolysis (Hamanaka and Mutlu, 2017). Later, these cells and their mitochondria are activated, inducing the differentiation process and proliferation of keratinocytes (Hamanaka and Mutlu, 2017). Merkel cells and melanocytes need healthy mitochondria to perform their roles in the skin. Mitochondrial malfunction malfunction leads to reactive oxygen species (ROS) production mecanosensoring decrease, decrease melanin synthesis, and senescence (Eckhart et al., 2019; Gu et al., 2020). Mutations in mitochondrial repair genes and haem production have been associated with several skin aberrations such as lipomas and pigmentation disorders (Stout and Birch-Machin, 2019). Wound healing and hair growth are also part of the physiological functions of the skin where mitochondria support the optimal regeneration and differentiation, respectively (Stout and Birch-Machin, 2019).

In the skin and other organs, damage in mtDNA increases with age (Figure 1B). Specific mutation patterns have been reported for skin cell mitochondria DNA, or more specifically, for its promoter (T414G transversion). This mutation has been also found in other aging tissues, such as the muscle and colon. Studies on skin samples have shown that mtDNA damage is correlated with oxidative stress, especially in exposed skin areas to UVR, such as the face and hands (Hudson et al., 2016). In these areas of increased sun exposure, Ray et al. found more epidermal skin mtDNA deletions in comparison to other less exposed areas. Furthermore, aged human fibroblasts have shown a raise in point mutations, meaning that different forms of mtDNA damage contributes to skin aging (Michikawa et al., 1999). The increase of 4977bp and 3895 bp deletion have been associated with skin age, it has been observed in the dermis of older individuals with excessive sun exposure (Eshaghian et al., 2006; Powers et al., 2016). As documented by several studies, the oxidative stress induced by UVR [Ultraviolet Radiation Type A (UVA) and Ultraviolet Radiation Type B (UVB)] produces alterations in the dermis, specifically in the DNA. The damage increases the mitochondrial dysfunction which will produce more ROS (Figure 1B) (Hudson et al., 2016). In consequence, many studies take the mtDNA as a skin aging marker, typically caused by UVR.

Time and external factors definitely affect the mitochondria and mtDNA (Figure 1B). It has been shown that hydrocarbons such as cigarette smoke and particulate matter (PM) are toxic to mitochondria and increase ROS production (Meyer et al., 2013). The information presented in this article and in other studies highlights that besides the increase in mtDNA mutations, there is a decrease in its content producing dysfunctions in the oxidative phosphorylation process. Interestingly, modifying the mtDNA polymerase γ (POLG1) in a mouse induced the mtDNA depletion mimicking the phenotypic changes related to aging and less activity of OXPHOS complex. The first changes were related to hair loss (alopecia), wrinkles, hyperkeratotic and hyperplastic epidermis, dermal inflammation, and acanthosis (Singh et al., 2018). These characteristics are comparable to aged human skin, which has leathery, increased laxity, uneven pigmentation, and brown spots (Hudson et al., 2016). After restoring mtDNA (Singh et al., 2018), mice have been shown to recover most of its normal phenotype. With this information, the possibility to replenish the defective mitochondrial content of aged cells in the skin by AMT/T could represent a viable option to restore healthy mtDNA copies and new functional mitochondria.

## The AMT/T Technique

The mitochondria transfer between cells has been studied *in vitro* and *in vivo* and associated with cell repair or improvements in cellular physiology (Paliwal et al., 2018). Among all cells that have the capacity to transfer mitochondria, mesenchymal stem/stromal cells (MSCs) have shown to induce remarkable properties by this process. Mesenchymal stem/stromal cells are able to transfer mitochondria to different cell types, including damaged cells, cancerous cells, and lung cells. They have shown positive effects reflected in cell repair, gain of function, and improving the cell’s overall energetics (Islam et al., 2012; Caicedo et al., 2015). In another example, astrocytes are able to exchange mitochondria as a key factor for neuron survival in a mouse model. After stroke, injured neurons release mitochondria, which are collected and recycled by astrocytes. In return, astrocytes share healthy mitochondria to neurons in order to enhance their recovery (Hayakawa et al., 2016). Cells naturally exchange mitochondria as repairing mechanisms. This process has inspired the development of new therapies, some of which have been developed by several researchers and are currently used *in vitro*, *in vivo*, and in clinical applications (Emani et al., 2017; Gollihue et al., 2018; Cabrera et al., 2019).

The use of mitochondria as a therapeutic agent by artificial mitochondria transfer/transplant (AMT/T) is gaining more evidence of its regenerative effects from *in vitro* to *in vivo* approaches, treating ischemic diseases in the majority of cases (Caicedo et al., 2015, 2017; Emani et al., 2017; Cabrera et al., 2019). This process aims to use mitochondria to repair damaged cells and tissue, alone or as a complement to current standards of therapy. AMT/T could be applied to enhance proliferation, migration, tissue regeneration and stress resistance of recipient cells (Caicedo et al., 2015; Lin et al., 2015; Emani et al., 2017; Cabrera et al., 2019). *In vitro*, Clark and Shay (1982) were the first to show that AMT/T is able to effectively transfer antibiotic resistance coded by the mtDNA to sensitive mammalian cells when they received exogenous mitochondria (King and Attardi, 1988). King and Attardi (1988) showed that reinjecting isolated mitochondria in mitochondria depleted mammalian cells can restore energy production based on respiration.

Since the first assays of AMT/T, different approaches have been developed in order to improve and simplify this process. Among the most common, are those related to chemical agents or physical adaptations to facilitate the transfer (Kitani et al., 2014; Lin et al., 2015; Kesner et al., 2016; Chang et al., 2017; Cabrera et al., 2019). Most of these methods are restricted to *in vitro* applications, however, it could be advantageous to transfer mitochondria to specific cells (*ex vivo* AMT/T) and reintroduce them back in the organism. Additionally, it could be a viable option to first isolate mitochondria and then modify them by coating or encapsulating them within membranes or vesicles. This could improve their internalization when applied in situ or systemically to treat an affected tissue.

As mitochondria have an important role in cell survival, transferring or transplanting them from healthy cells or tissue to damaged sites in the body has emerged as a rising therapeutic option. McCully’s team in 2018 was the first to use mitochondrial transplantation on human beings. His team transplanted respiratory competent mitochondria into ischemic injured tissues in pediatric patients with myocardial dysfunction. Indeed, ischemia-reperfusion induces loss of viable mitochondria, decreases ATP production, and leads to myocardial hypoxemia and necrosis. McCully and his team treated five children, three females and two males of different ages (4 days, 6 days, 25 days, 6 months and 2 years). All of them were suffering from congenital cardiac malformations and during surgery they endured ischemia-reperfusion damages requiring extracorporeal membrane oxygenation (ECMO), which is a mechanical circulatory support. Children were eligible for mitochondrial transplantation if they already had a recent cardiac surgical procedure preceding an acute onset of coronary artery obstruction. During a second intervention, they were injected with isolated autologous healthy mitochondria directly into the myocardium affected by ischemia which was identified by echographic hypokinesis. The outcome was an improvement of global cardiac function, regional myocardial hypokinesis, and mortality. Four patients improved their left ventricular function, but two out of five died: the first, despite myocardial function improvement, showed failure in other organs and the second one died because of respiratory issues. Given the small number of patients and lack of a control group, it is difficult to attribute the effects of improvement in ventricular function to mitochondrial transplantation (Emani et al., 2017; Emani and McCully, 2018). However, their study shows that this technique may be expanded to treat other forms of myocardial ischemic injuries or organs damaged by ischemia-reperfusion. It is also recommended a prospective clinical trial to assess the safety, efficacy, and optimal dosing of mitochondria for clinical use (Emani et al., 2017; Emani and McCully, 2018).

## AMT/T Applications to Treat Tissue Damage

Artificial mitochondria transfer/transplant has so far shown positive effects in cellular and tissue repair, and recent literature highlights its potential medical use. Many types of diseases or damaged associated conditions could be treated with AMT/T. Most of them are related to ischemic events, metabolic conditions, aging degeneration, and inflammatory associated diseases (Lin and Beal, 2006; Islam et al., 2012; Masuzawa et al., 2013; Emani et al., 2017). Conditions involving cardiovascular diseases treated with AMT/T *in vivo*, *in vitro*, and in humans have been widely studied (McCully et al., 2009; Masuzawa et al., 2013; Kaza et al., 2017; Emani and McCully, 2018). Other promising therapeutic targets of AMT/T are neurodegenerative diseases, spinal cord injury (Kang et al., 2016), and mitochondria hereditary conditions (Chang et al., 2016; Gollihue and Rabchevsky, 2017; Gollihue et al., 2018). AMT/T’s ability to repair damaged cells and renew the mitochondrial pool has changed the view of the mitochondria as a powerhouse of the cell to a therapeutic agent and a key element in regenerative medicine.

Important advances have been evidenced, especially *in vivo*, regarding the application of AMT/T after cardiovascular ischemic events with healthy autologous or allogeneic mitochondria. These assays have shown that AMT/T repairs the damage in the affected tissue and favors the recovery of mitochondria functions (McCully et al., 2009; Masuzawa et al., 2013; Kaza et al., 2017; Shin et al., 2017; Emani and McCully, 2018). Most of the time, ischemic cardiovascular diseases are the result of a lack of oxygen carrying blood reaching tissues. Ischemia alters mitochondrial function inducing ROS generation, decreasing ATP production, promoting cellular stress, and leading to apoptosis (Chen et al., 2011; Kalogeris et al., 2014). AMT/T could be a promising cardioprotective or therapeutic treatment, if injected into infarcted areas. For example, it has been observed that AMT/T could prevent ischemia-reperfusion injury. Autologous mitochondria injections in ischemic induced lesions in rabbits’ hearts enhanced oxygen consumption, cell viability, and post-infarct cardiac function. It was shown that transplanted mitochondria are quickly internalized by cardiac cells, helping to restore normal energy synthesis (Masuzawa et al., 2013).

Other studies showed benefits of mitochondria transfer in heart ischemia *in vivo*. Myocardial ischemia was induced in pigs where autologous mitochondria were injected directly into the ischemic region for the tested group while the control group did not receive any treatment, only a vehicle. Assessment of the ischemic markers showed a significant increase in cardiac troponin I and creatine kinase levels in the control compared to the tested group, suggesting a better post-ischemic myocardial function after AMT/T (Cowan et al., 2016; Kaza et al., 2017). It has been shown that the delivery of mitochondria through the coronary vasculature protects the ischemic myocardium. As part of this study, rabbits’ hearts were exposed to 30 min of ischemia and then 10 and 120 min of reperfusion. Mitochondria were either injected in the ischemic region or delivered by vascular perfusion through the coronary arteries at the beginning of the reperfusion procedure. Mitochondria were found near the site of delivery in the in situ administration, however, vascular perfusion had better dispersion through the heart and better protective effects after 10 and 120 min reperfusion (Cowan et al., 2016).

Heart transplant is a procedure that involves keeping a donor’s heart preserved outside the body until it is received by the patient. During the process the donor’s heart passes, in the first instance, through cold ischemia time (CIT), which is about 4–6 h for humans. Cold ischemia time starts after cutting circulation and putting the organ in a cold transport solution. Then the transplanted heart is connected to the patient’s circulation where ischemia reperfusion (IR) can cause injury as well. It has been shown that CIT causes IR injury to the heart mitochondria affecting myocardial function and tissue viability. Heterotopic heart transplantation was performed in mice and AMT/T was delivered in the coronary arteries before and after CIT. The experiments showed that mitochondrial transplant enhances heart function, decreases tissue injury, and reduces cold graft failure, thus improving the transplantation success after prolonged CIT (Moskowitzova et al., 2019).

Stroke or “brain attack” is a neurovascular disease whose characteristics and consequences could be treated with AMT/T. A decreased blood flow induces ischemia in specific regions of the brain, leading to oxygen and nutrient deprivation and causing tissue death. Mitochondria function and dynamics are particularly damaged, especially regarding its capacity to maintain the respiratory chain proton gradient and produce ROS. This leads to further structural damage, apoptosis, cell degradation, and inflammation (Sims and Anderson, 2002; Niizuma et al., 2010; Yang et al., 2018). Stroke can affect specific brain areas, damaging memory, muscle control, or inducing visual loss (Siesjö, 1992; Hossmann, 2006; Teasell and Hussein, 2013). Testing the possibility to repair the loss of healthy mitochondria due to stroke by AMT/T has shown so far to improve recovery in preclinical assays. It has been observed that the use of xenogeneic mitochondria from hamsters to treat acute injurious ischemic stress in the central nervous system by AMT/T resulted in enhanced neuronal survival and recovery of motor activity (Huang et al., 2016).

Neurodegenerative diseases including Parkison’s, Alzheimer’s, and Huntington’s are characterized by the progressive loss of neurons and induced death related to protein aggregation and inclusion body formation in cells (Ross and Poirier, 2004; Krols et al., 2016). Together with these factors, mitochondria dysfunction seems to play an important part in the progression of these diseases. Parkinson’s disease (PD) is mainly due to a progressive degeneration of dopaminergic neurons, located in the substantia nigra (Moore et al., 2005; Chaudhuri and Schapira, 2009). Lack in dopamine secretion causes symptoms such as tremor, akinesia, and muscular hypertonia. Parkinson’s progression is linked to misfolding and aggregation of α-synuclein. It has been reported that a type of α-synuclein aggregate, smaller and distinct in its conformation, called pα-syn^∗^, is particularly affecting mitochondria, causing its damage and mitophagy (Grassi et al., 2018). As mitochondria is affected in Parkinson, it has been proposed to treat the disease with AMT/T. It has been observed in a PD-mice model that AMT/T led to an increase in the striatal mitochondrial activity and ATP production, a decrease of ROS levels, and improvements in Parkinson’s symptoms (Shi et al., 2017). Parkinson’s disease-induced rats treated with AMT/T showed that allogeneic mitochondria transplantation improves their motor activities, and pointed out a substantial decrease in dopaminergic neuron loss (Chang et al., 2016). Understanding how AMT/T can improve PD could open new treatments for this disease including using it together with current therapeutic options. Parkinson’s disease is not the only neurodegenerative pathology that could be treated by AMT/T. Literature also discussed the role of mitochondria in Huntington disease, Alzheimer’s disease, Amyotrophic Lateral Sclerosis, and Friedreich’s Ataxia, highlighting the potential application of AMT/T in rare neurodegenerative pathologies (Lin and Beal, 2006; Kim et al., 2010; Carvalho et al., 2015). There is a scientific and clinical need to perform more studies where AMT/T could be used as a treatment for neurodegenerative diseases.

The exposure to toxic substances could induce liver mitochondrial dysfunction, leading to a diverse array of acute or chronic injuries (Deavall et al., 2012; Gu and Manautou, 2012). Acetaminophen-induced liver injury causes mitochondrial oxidative stress, worsening the disease (Yan et al., 2018). Toxins, such as ethanol and a variety of drugs, can impair beta-oxidation directly or through affects mitochondrial function. Mitochondrial dysfunction leads to abnormal permeability transitions, initiating hepatocyte apoptosis and necrosis (Pessayre et al., 1999; Shi et al., 2018). AMT/T was used to treat the damage induced by Acetaminophen liver failure by paracetamol intoxication. Mitochondria were isolated from human hepatic cells and injected intravenously in mice injured liver. After mitochondria administration, transaminase activity significantly decreased, indicating that exogenous mitochondria were protecting cells against toxicity. Mitochondria spread in several tissues and increased hepatocyte energy production, reduced ROS levels, and improved tissue regeneration (Fu et al., 2017; Shi et al., 2018). This study suggests that AMT/T may prevent acetaminophen-induced liver injury.

From the first assays of AMT/T by Clark and Shay in 1982 to the clinical application of mitochondria by McCully’s team in 2018, there is strong evidence showing that receiving exogenous healthy mitochondria by damaged cells or tissues help them to recover (Clark and Shay, 1982; Caicedo et al., 2017; Emani et al., 2017). Not only does AMT/T improve tissue recovery but it also has been shown to promote immune regulation. It has been observed that syngeneic or allogeneic mitochondria delivered by intraperitoneal injection in mice does not cause an immunogenic reaction, thus opening the possibility to use mitochondria from different donors to heal damaged tissue (Ramirez-Barbieri et al., 2019). It has been shown that AMT/T has important intracellular consequences leading to short-term improvement of bioenergetics and a “supercharged” state. However, the resulting state disappears over time (Ali Pour et al., 2020). These work together with the use of AMT/T to turn TH17 cells into Tregs promoting an immune regulatory effect. Also, the use of PAMM to repair the UVR damage of mitochondria supports the hypothesis of the possible use of mitochondria, from different donors, like PAMM to treat skin aging.

## AMT/T for the Antiaging Use of Mitochondria

The skin is a mixture of different layers of cells working together with the immune, nerve, and vascular systems. Aging affects all skin components differently, but the transfer of healthy mitochondria could be a viable option to repair skin cell senescence and loss of function. Skin contains sets of cells with different viability during time and energy consumption. Epidermal keratinocytes stem cells and other stem cells have a long period for permanence in skin and are very susceptible to damage (Panich et al., 2016; Eckhart et al., 2019). Keratinocytes are cells that have a short lifespan and high metabolic activity and needs (Eckhart et al., 2019). Cells such as Merkel and melanocytes are long-lived and have a high metabolic activity, prompting them to accumulate damage with time (Figure 1B) (Eckhart et al., 2019). Among these cells, fibroblasts, keratinocytes, immune cells (such as Langerhans, mechanoreceptors), as merkel cells and melanocytes, are all susceptible to aging and its damaging effects (Quan et al., 2015; Blatt et al., 2017; Tobin, 2017; Kaddurah et al., 2018). However, all these cells have the potential to internalize mitochondria and be repaired. It has been shown that cells *in vitro* and *in vivo* are capable of uptaking mitochondria by different and active mechanisms like macropinocytosis and actin dependent internalization (Kitani et al., 2014; Pacak et al., 2015). Once inside, it seems that isolated mitochondria fuse with the endogenous mitochondria by the activity of mitofusins MFN1, MFN2 on the outer membrane and OPA1 on the inner membrane (Alavi and Fuhrmann, 2013; Cowan et al., 2017). The understanding of the energetic and survival fate of exogenous mitochondria inside the recipient cells is moving forward, however, important questions are still unanswered. So far the energetic boost and possibly renewal of the endogenous mitochondria pool could explain part of transfer/transplant regenerative properties (Ali Pour et al., 2020).

It has been reported that keratinocytes and fibroblasts accumulate mtDNA mutations after UVR exposure, increasing its biological aging and stress (Figure 1B) (Koch et al., 2001; Birket and Birch-Machin, 2007; Paz et al., 2008; Cazzato et al., 2018; Parrado et al., 2019). These cells could internalize mitochondria, repairing their viability and providing an “energetic boost” in the basal membrane (Koch et al., 2001; Ali Pour et al., 2020). The energetic boost could have positive effects on the fibroblasts and keratinocyte’s proliferation and migration. Less Langerhans cells are present in aged skin where a cascade of regulatory and proinflammatory cytokines takes place (Kabashima et al., 2003; Bocheva et al., 2019). Ultraviolet radiation induces immunosuppression and tolerance on Langerhans cells, due the presence of TNF-a, prostaglandin PGE2, and IL-10 (Parrado et al., 2019). However, a great quantity of ROS is induced by UVR, resulting in skin photoaging and inflammation (Pillai et al., 2005; Awad et al., 2018). The mitochondria transfer to immune cells by PAMM or by the isolation of MSC’s mitochondria repairs UVR damage and changes the cells from proinflammatory to immune regulatory (Cabrera et al., 2019; Luz-Crawford et al., 2019). The interaction between mitochondria and immune cells in the skin is puzzling as it could induce an immune regulatory effect in Langerhans cells, however, it would help them to survive and proliferate. Mitochondria transfer/transplant to nerve cells has shown to have effects such as improving their activity, growth and viability (Chang et al., 2019). When Merkel cells are exposed to the sun, their cellular density increases in a process that accumulates with age, leading to carcinogenesis, especially in elderly white men (Moll et al., 1990; Coggshall et al., 2018). A loss in the perception, fromsoftouch to itching has been associated with aging (alloknesis). This problem is correlated with a low number of merkel cells (Feng et al., 2018). Merkel cells have a long-term permanence in the skin where the AMT/T would help them to stay viable for a longer period of time (Eckhart et al., 2019) (Figure 1B).

Melanocytes interact and influence keratinocytes metabolism through melanosomes transfer. Melanocytes affected by aging play a crucial role in disseminating damaging factors among cells in the skin. Melanogenesis is a multistep process regulated by melanocortins (MSH) and adrenocorticotropins, leading to the control of the tyrosinase enzyme using L-tyrosine as a precursor (Slominski et al., 2004; Slominski A. et al., 2012). L-tyrosine is also a precursor of thyroid hormones which in turn regulate melanin pigmentation (Slominski et al., 2004). There exist two major melanine variants: pheomelanin (red head and freckles) and eumelanin (dark haired individuals), depending on the MCR1 (melanocortin 1 receptor) gene regulation (Stout and Birch-Machin, 2019). The first has been associated with a higher prooxidant effect because its synthesis consumes cysteine and glutathione antioxidant (Birch-Machin and Bowman, 2016). In this context, it has been hypothesized that stimulation of eumelanin has a protective effect on mtDNA copy number (Stout and Birch-Machin, 2019). However, interactions between mtDNA and pheomelanin/eumalin ratio have not been properly researched. It has been shown that Melanocytes are able to express senescence markers such as p16INK4A, HMGB1, in addition to having dysfunctional telomeres. Interestingly, aged melanocytes can induce telomere dysfunction and senescence in surrounding cells via CXCR3 activation and ROS production (Victorelli et al., 2019).

Melanocytes are sensitive to oxidative stress due to the pro-oxidant state induced during melanogenesis (Denat et al., 2014; Slominski and Carlson, 2014). This process is confined to the melanosomes, which protects the cell from oxidative damage (Denat et al., 2014). Interestingly, melanosomes switch from oxidative catabolism to anaerobic glycolysis, activating the pentose phosphate pathway in the keratinocytes and possibly protecting them from oxidative damage (Slominski et al., 1993, 2014, 2015b). It has been observed that Alpha-melanocyte-stimulating hormone (alpha-MSH) stimulates melanogenesis, thus leading to the protection of keratinocytes from UVR induced DNA and mtDNA damage (Böhm and Hill, 2016). Mitochondria in melanocytes interact with melanosomes suggesting an induction of melanosome biogenesis and melanin production (Naidoo et al., 2018; Stout and Birch-Machin, 2019). Melanin protects cells and their mtDNA from stressors such as UVA, UVB and H_2_O_2_ (Swalwell et al., 2012). Both the reduction of aerobic metabolism and the protection of mtDNA damage by melanosome transfer from melanocytes to keratinocytes could prevent accelerated skin aging. It is possible that renewing the mitochondrial pool in both cells could have beneficial effects in reducing the damage of UVR and environmental factors.

Melatonin is a hormone product of the pineal gland that regulates the circadian rhythm. It has been observed that melatonin can be produced by epidermal and hair follicle keratinocytes (Kleszczynski and Fischer, 2012; Slominski et al., 2020, 2008). Melatonin has ROS scavenging properties it promotes antioxidant and DNA repair mechanisms, inducing immune modulation and antitumor properties (Slominski et al., 2018a). Interestingly, melatonin can be transferred to mitochondria by the peptide transporter PEPT1/2. This improves the mitochondrial membrane potential by inhibiting the activation of the permeability pore, stimulating the activity of uncoupling proteins (UCPs), and inducing an increase in ATP production (Slominski et al., 2018a). Melatonin and its derivatives N1-acetyl-N2 -formyl-5-methoxykynurenine (AFMK) and N-acetyl serotonin (NAS) are expressed in the human skin and have shown antioxidant and antiapoptotic activity (Skobowiat et al., 2018; Slominski et al., 2020). The secretion of melatonin by the pineal gland declines with age (Hardeland, 2012) and possibly affects the presence and production of melatonin in the skin. Melatonin strongly interacts with mitochondria, protecting them from UVR, ROS, and aging damage. Mitochondria are also able to produce melatonin in the mitochondrial matrix (Hardeland, 2012; Tan et al., 2016). Understanding the relationship between melatonin, its derivatives and AMT/T is key to mitigating the loss of skin functionality during the aging process (Figure 1B).

Mitochondria may also interact with melanocyte proliferation and tyrosinase activity as *in vitro* experiments have shown their inhibition in the presence of melatonin, a hormone that heavily relies on mitochondria and ROS signaling (Stout and Birch-Machin, 2019). This is how mitochondrial damage may contribute to an alteration of skin pigmentation and aging. Melanocyte degeneration, cell cycle arrest, and apoptosis have been associated with oxidative stress due to mitochondrial dysfunction in Vitiligo (Tang et al., 2019; Yi et al., 2019).

Mitochondria, as a modulator of skin pigmentation, play an important role in the initial protection against UVR (Stout and Birch-Machin, 2019). More pigmentation (more melanin) is inversely related to DNA lesions in humans. mtDNA damage is vastly seen in photoexposed skin, leading to cellular damage and death (Stout and Birch-Machin, 2019). Even though aging would eventually happen due to the shortening of the telomere caps in nuclear DNA and consequent senescence, UVR is a major key factor in aging pathogenesis. This “photoageing” needs to be clarified as many aspects have been studied over the years: collagen, mtDNA damage, increased ROS production, and others (Birch-Machin and Bowman, 2016). Ultraviolet radiation causes inflammation that breaks down matrix proteins and the activation of the ROS signaling pathways. This activates NFkB and AP-1, which inhibits collagen formation while old collagen is being destroyed. mtDNA damage in photoexposed areas increases ROS production and may accelerate skin aging (Stout and Birch-Machin, 2019). Skin exposed to UVR causes deletions of mtDNA, increased up to tenfold, compared with sun-protected skin of the same individuals (Berneburg et al., 1999). In human skin, UVR-induced deletions were found to persist for years and their levels increased after cessation of UVR irradiation even in the absence of further exposures (Krutmann and Schroeder, 2009; Krutmann, 2017). As was mentioned before, there are differences in the composition of extracellular matrix of papillary and reticular dermis. They differ also in rate of cell division, contraction, and in expression of various collagens and proteoglycans. Fibroblasts are responsible for synthesizing all types of fibers and ground substance, but their function depends on the specific tissue compartment where they reside (Lynch and Watt, 2018).

Majora et al. (2009) seeded human skin fibroblasts into collagen gels to generate dermal equivalents. These cells were either derived from Kearns-Sayre syndrome patients, who constitutively carry large amounts of the UVR-inducible mitochondrial common deletion, or normal human volunteers (Majora et al., 2009). Human skin fibroblasts carrying large amounts of the UVR-inducible common deletion in their mitochondrial genome translate functional and structural alterations into the dermal equivalent.

Ultraviolet radiation damages skin cells, especially their genetic material, leading to cancer (Rybchyn et al., 2018; Singh, 2019). Furthermore, UVR negatively affects mitochondria structure and function, thus leading to the generation of ROS and decreased ATP production (Rybchyn et al., 2018). Interestingly, it has been reported that 1α,25-Dihydroxyvitamin D3 (1,25(OH)2D3) induces an energy-conserving mechanism, which reduce UVR-DNA damage and skin carcinogenesis (Rybchyn et al., 2018; Chaiprasongsuk et al., 2019). This mechanism works by increasing glycolysis in the affected cells, arresting mTORC1 and growth, activating autophagy and inhibition of proliferation, and repairing mitochondria (Rybchyn et al., 2018). This evidence could lead to a treatment of UVR harmed skin that includes the administration of mitochondria mixed with 1,25(OH)2D3. If 1,25(OH)2D3 is inducing an energy-conserving mechanism, it would be possible to use the AMT/T after the autophagic mechanism, to help cells restore their mitochondrial patrimony and even mix with the transferred mitochondria.

In several reports, human dermal fibroblasts in tissue culture showed that carbonylated proteins, a marker for severe and chronic oxidative stress, were elevated in the last third of life (Stadtman and Levine, 2000). Disturbance in the prooxidant-antioxidant balance causes oxidative stress that is associated with aging and senescence. ROS are mainly produced by mitochondria during normal metabolism. High ROS levels produced by dysfunctional mitochondria have been suggested as the main cause of aging (Barja, 2014).

Intrinsic changes in the aged fibroblast, as well as exposure to environmental insults, lead to a progressive increase in connective tissue damage. This is mediated by matrix metalloproteinases (MMPs) and a reduction in new collagen synthesis. As the connective tissue becomes progressively damaged, it eventually becomes a stimulator of reduced fibroblast synthetic function.

The fibroblast growth factor (FGF) has a relevant role in anti-aging therapy as it is related to collagen and elastin synthesis activation, which is responsible for skin resistance and elasticity, characteristics that are diminished with skin aging (Maddaluno et al., 2017). The progressive decline in the antioxidant capacity associated with age results in increased production of reactive oxygen species from oxidative metabolism in skin cells (Fabi and Sundaram, 2014), and diminished mitophagy (Höhn et al., 2017) could be treated by AMT/T possibly in combination with other factors such as FGF.

Hair quality may be also considered as a marker of aging. Melanocytes and keratinocytes determine hair color and growth. It has been hypothesized that mtDNA damage increases oxidative stress in the context of an acquired base pair deletion and is responsible for hair graying (Stout and Birch-Machin, 2019). Hair loss is a marker of aging and may be related to nutrient deficiency, alopecia, and androgen stimulation. In non-balding regions, there is an increased cytoplasmic and mitochondrial expression of superoxide dismutase (Stout and Birch-Machin, 2019). This represents a protective mechanism that promotes oxidative resistance and a repair function. Although very important questions need to be answered regarding AMT/T, hypothesizing that isolated mitochondria could be internalized by melanocytes to help cells produce energy more efficiently, less ROS (Zhang et al., 2019), and ultimately fewer mutations are plausible.

It is our belief that the procedure for delivering mitochondria by AMT/T to the skin and its cells is key to inducing its repair mechanisms. The kind of mitochondria, their origin, and if they have been modified in any way should be considered when evaluating its regenerative capacities (Caicedo et al., 2017; Miliotis et al., 2019). After defining determining the best mitochondria, one method of delivering them could be to transfer it ex vivo to patient’s skin cells by MitoCeption and reintroducing them in the affected area (Caicedo et al., 2015, 2017; Cabrera et al., 2019; Miliotis et al., 2019). Delivering mitochondria with needles, especially microneedles (Sun et al., 2015), could be an option, however, special attention should be made as skin is very thin in some areas and it is important to traverse the stratum corneum (Kochhar et al., 2013). Microneedle patches have shown to be painless and easy to use, with modulable parameters desirable for drug delivery (Prausnitz, 2017). Microneedle patches could be designed for mitocondria delivery and tested together with *in vivo* experiments. As previously mentioned, mitochondria doesn’t seem to need encapsulation or additives to be uptaken by cells, however, how mitochondria is going to be suspended or if an emulsion or microemulsion (Hajjar et al., 2017) is needed to traverse the stratum corneum, will need further research (Figure 1B).

## Conclusion

Environmental factors besides time can induce the acceleration of aging and the mitochondrial dysfunction of skin (Panich et al., 2016; Victorelli et al., 2019; Gu et al., 2020). Exposure to these factors, such as sunlight, could be prevented by sunscreens or simply by limiting the exposition to UVR. However, they are difficult to repair once damage is present (Hudson et al., 2016). Even if mitochondrial dysfunction is mitigated through intracellular mechanisms, such as the quality control process (Suliman and Piantadosi, 2016), damages still accumulate with time, decreasing the overall life-span of a person (Martínez-Cisuelo et al., 2016; Nacarelli et al., 2016). Based on these observations, it is plausible to propose AMT/T as a way to enrich the endogenous pool with healthy mitochondria, even if questions about intracellular interactions and transfer effects still remain today (Ali Pour et al., 2020).

Skin function relies on many cellular and intracellular mechanisms, among them mitochondria integrity. Many of the mechanisms contributing to skin aging are not fully understood; nevertheless, it is clear that mitochondria play an important role within a complex network (Birket and Birch-Machin, 2007; Hudson et al., 2016; Gu et al., 2020). Among them, mitochondria metabolism of substances like melanin maintain intracellular homeostasis by photoprotective processes (Naidoo et al., 2018; Tang et al., 2019). As a result, the accumulation of small abnormalities or deficits may lead to larger negative effects on the skin and its components if cells endogenous mitochondria is not treated.

The skin is a complex tissue of layers and cells that play different functions (Tobin, 2017). Skin cells and structures suffer differently after being exposed to aging inducing factors such as UVR, where mitochondria are one of the most affected organelles (Hudson et al., 2016). As seen in literature, the AMT/T is able to repair damage, especially hypoxia, in many cells and tissues (Berridge et al., 2016; Torralba et al., 2016; Caicedo et al., 2017; EP3169338A1, 2019; – Methods for the intercellular transfer of isolated mitochondria in recipient cells – Google Patents). AMT/T has shown regenerative effects *in vitro* and *in vivo*. Isolated mitochondria could be an interesting therapeutic agent of choice to neurodegenerative and cardiovascular diseases (McCully et al., 2016; Boukelmoune et al., 2018). Here we propose the hypothesis that AMT/T would help the skin and its cells to persist or recover after aging inducing factors exposition and be viable for a longer alone or as a complement to current standards of care and prevention.

Still many challenges need to be overcome in order to generalize the application of mitochondria as a therapeutic agent to accelerate aging and disease. What is the effect of isolated mitochondria from different tissues on different tissues, do they behave the same. Different methods of administration must be tested in order to find the best for each application (Cowan et al., 2016; Ramirez-Barbieri et al., 2019). How to protect the isolated mitochondria during the process of transfer/transplant, are there mitochondria that are more resistant than others as it seems that some can normally exist outside cells without a cover membrane (Al Amir Dache et al., 2020; Bertero et al., 2020; McCully et al., 2020). This article intends to present one of the many aspects in which mitochondria could be used as an universal treatment for cell damage and aging.

## Author Contributions

MB and SC provided substantial and equal contributions to the manuscript regarding the acquisition of information, analysis, and interpretation. TB and PP provided significant and equal contribution to the manuscript, acquisition, and interpretation of data. SB, EC, AB, and CC provided information, interpretation, and analysis. FC and RD provided a substantial contribution to the manuscript, data acquisition, interpretation of information, supervised the work and mentorship, and provided financial support for the cooperation. AC conceptualized the article, and the research goals and statements, provided a substantial contribution to the manuscript, data acquisition, analysis of the information, supervised the work, mentorship of the team, and funding acquisition. All authors contributed to drafting the work and revising it critically for relevant intellectual content, and final approval of the last version to be published.

## Conflict of Interest

The authors declare that the research was conducted in the absence of any commercial or financial relationships that could be construed as a potential conflict of interest.
